# Study on the semi-supervised learning-based patient similarity from heterogeneous electronic medical records

**DOI:** 10.1186/s12911-021-01432-x

**Published:** 2021-07-30

**Authors:** Ni Wang, Yanqun Huang, Honglei Liu, Zhiqiang Zhang, Lan Wei, Xiaolu Fei, Hui Chen

**Affiliations:** 1 School of Biomedical Engineering, Capital Medical University, No.10, Xitoutiao, You An Men, Fengtai District, Beijing, 100069 People’s Republic of China; 2 Beijing Key Laboratory of Fundamental Research on Biomechanics in Clinical Application, Capital Medical University, Beijing, 100069 People’s Republic of China; 3 Information Center, Xuanwu Hospital, Capital Medical University, Beijing, 100053 People’s Republic of China

**Keywords:** Patient similarity, Electronic medical records, Semi-supervised learning, *k*-nearest neighbors, Liver diseases

## Abstract

**Background:**

A new learning-based patient similarity measurement was proposed to measure patients’ similarity for heterogeneous electronic medical records (EMRs) data.

**Methods:**

We first calculated feature-level similarities according to the features’ attributes. A domain expert provided patient similarity scores of 30 randomly selected patients. These similarity scores and feature-level similarities for 30 patients comprised the labeled sample set, which was used for the semi-supervised learning algorithm to learn the patient-level similarities for all patients. Then we used the k-nearest neighbor (*k*NN) classifier to predict four liver conditions. The predictive performances were compared in four different situations. We also compared the performances between personalized *k*NN models and other machine learning models. We assessed the predictive performances by the area under the receiver operating characteristic curve (AUC), F1-score, and cross-entropy (CE) loss.

**Results:**

As the size of the random training samples increased, the *k*NN models using the learned patient similarity to select near neighbors consistently outperformed those using the Euclidean distance to select near neighbors (all P values < 0.001). The *k*NN models using the learned patient similarity to identify the top *k* nearest neighbors from the random training samples also had a higher best-performance (AUC: 0.95 vs. 0.89, F1-score: 0.84 vs. 0.67, and CE loss: 1.22 vs. 1.82) than those using the Euclidean distance. As the size of the similar training samples increased, which composed the most similar samples determined by the learned patient similarity, the performance of *k*NN models using the simple Euclidean distance to select the near neighbors degraded gradually. When exchanging the role of the Euclidean distance, and the learned patient similarity in selecting the near neighbors and similar training samples, the performance of the *k*NN models gradually increased. These two kinds of *k*NN models had the same best-performance of AUC 0.95, F1-score 0.84, and CE loss 1.22. Among the four reference models, the highest AUC and F1-score were 0.94 and 0.80, separately, which were both lower than those for the simple and similarity-based *k*NN models.

**Conclusions:**

This learning-based method opened an opportunity for similarity measurement based on heterogeneous EMR data and supported the secondary use of EMR data.

## Background

In recent years, the sharp increment in electronic medical records (EMRs) adoption has facilitated the move toward personalized medicine [1–3]. The wide use of EMR opened opportunities in making a personalized decision for a given patient [4]. Especially, EMR data for the similar patients in conjunction with machine learning algorithms was applied to build individualized models to predict the risk for a potential disease [5–7] or to identify the risk factors [8, 9] and the disease subgroups [10, 11], showing an improved performance in these tasks.

EMR data is usually heterogeneous, making it more difficult than common homogeneous data to measure the patient similarity for further use. Many non-numeric features, such as diseases and procedures, that are important and informative in assisting medical clinicians in making a diagnostic decision. Additionally, symptoms and signs are also useful that are often recorded in a free-text form. Generally, these features cannot be input into some similarity- or distance-based machine learning models with numeric features due to the difficulty in computing a uniform similarity or distance for features with different data types. For example, the widely used Euclidean distance was effective in measuring the distance among continuous variables but not among discrete variables. Moreover, traditional distance measurements did not consider some hierarchical code systems’ ontological distance. To address these problems, some researchers calculated feature similarities respectively and then integrated them into a single value with weighted sum [5] or geometric mean [3, 12]. However, the parameters used and their settings in integrating feature similarities into a single patient similarity may depend more on subjective judgment and lack conviction.

In this study, we proposed a new learning-based method to obtain patient similarity after the direct computation. We first calculated feature-level similarities by using different similarity measurements for different types of features. They were then input into a semi-supervised learning (SSL) algorithm to learn the patient-level similarities. The proposed patient similarity was validated by selecting similar samples to predict the status of liver diseases. Liver diseases were usually multifactorial complex with high global incidence [11] and had affected over one-fifth of the population in China [13]. The non-alcohol fatty liver disease (NAFLD) had been identified as an emerging health problem worldwide [14], and liver hemangiomas were the most common benign liver tumors occurring in people of all ages [15, 16]. Also, China had the 9th highest rate and the largest number of liver cancer patients in the world [17]. Thus, there is an urgent need to help clinicians make a proper diagnostic decision for a patient of these liver diseases. Consequently, the proposed patient similarity was applied in predictive models for three common liver diseases (liver cancer, hemangioma, and NAFLD), attempting to assist the clinicians in diagnosis.

To the best of our knowledge, it is the first time that the patient similarity was learned through an SSL algorithm using the calculated feature similarities. This method can be easily implemented in other tasks and is likely to be an important step in facilitating personalized medicine based on heterogeneous EMR data.

### Related work

Patient similarity has become a hot topic in recent years, with many researchers using patient similarity as a tool to enable precision medicine. For heterogeneous EMR data, two strategies were usually adopted when evaluating patient similarity. All features were standardized in advance for a static distance metric [18] in the first strategy. Lee et al. [19] used the cosine similarity metric to identify similar patients for the downstream 30-day mortality prediction based on the MIMIC-II database. All predictor variables were presented as a numeric vector to yield the cosine-based similarity metric. David et al. [20] utilized the Euclidean distance-based metric to select similar patients for anomaly detection and characterization on the basis of numeric laboratory data. Li et al. [10] assembled different types of variables into a numeric data matrix, with a one-hot representation for non-numeric ICD-9-CM codes. The cosine distance metric was then used to construct a patient-patient network for further disease subtype identification. Gu et al. [21] adopted a weighted Euclidean distance to evaluate similarity for both continuous and discrete variables. These studies either merely considered numeric variables [20, 21] or standardized all variables in advance for further patient similarity measurement [10, 19]. This strategy might result in feature information loss during standardization [18] and did not utilize all EMR data features.

Therefore, many researchers chose the second strategy to evaluate patient similarity for heterogeneous EMR data. A list of feature similarities was calculated for each data type separately. Wang et al. [5] built predictive models for diabetes’ risk prediction based on EMR data. Feature similarities were firstly evaluated according to variables’ attributes. The weighted sum of feature similarities was calculated to form a single patient similarity score, in which the weights were allocated by trial and error. Gottlieb et al. [3, 12] assessed feature similarities and integrated them into a single similarity score with a weighted geometric sum. Huang et al. [22] also calculated feature similarities separately and compared all the possible combinations of three types of disease code, three laboratory test sets, and three weight allocation schemes. Zheng et al. [23] used the supervised XGBoost algorithm to learn patient similarities. Pairwise similarities for symptoms, lab tests, and preliminary diagnoses were measured to serve as inputs of the XGBoost model, and those for discharge diagnoses as the output. For a new patient, the calculated feature similarities with all patients were given to the trained XGBoost model to predict the patient similarities for downstream discharge diagnoses prediction. In these studies, weights were allocated subjectively, leading to a lack of conviction to some degree. Besides, the supervised label and learning effect of the XGBoost-based method might vary with the similarity assessment method adopted for discharge diagnoses.

We proposed a semi-supervised learning method to cover all available EMR data features, automatically weight each feature similarity, and save time and labor costs for labeling simultaneously.

## Methods

### Overview

**Fig. 1:**
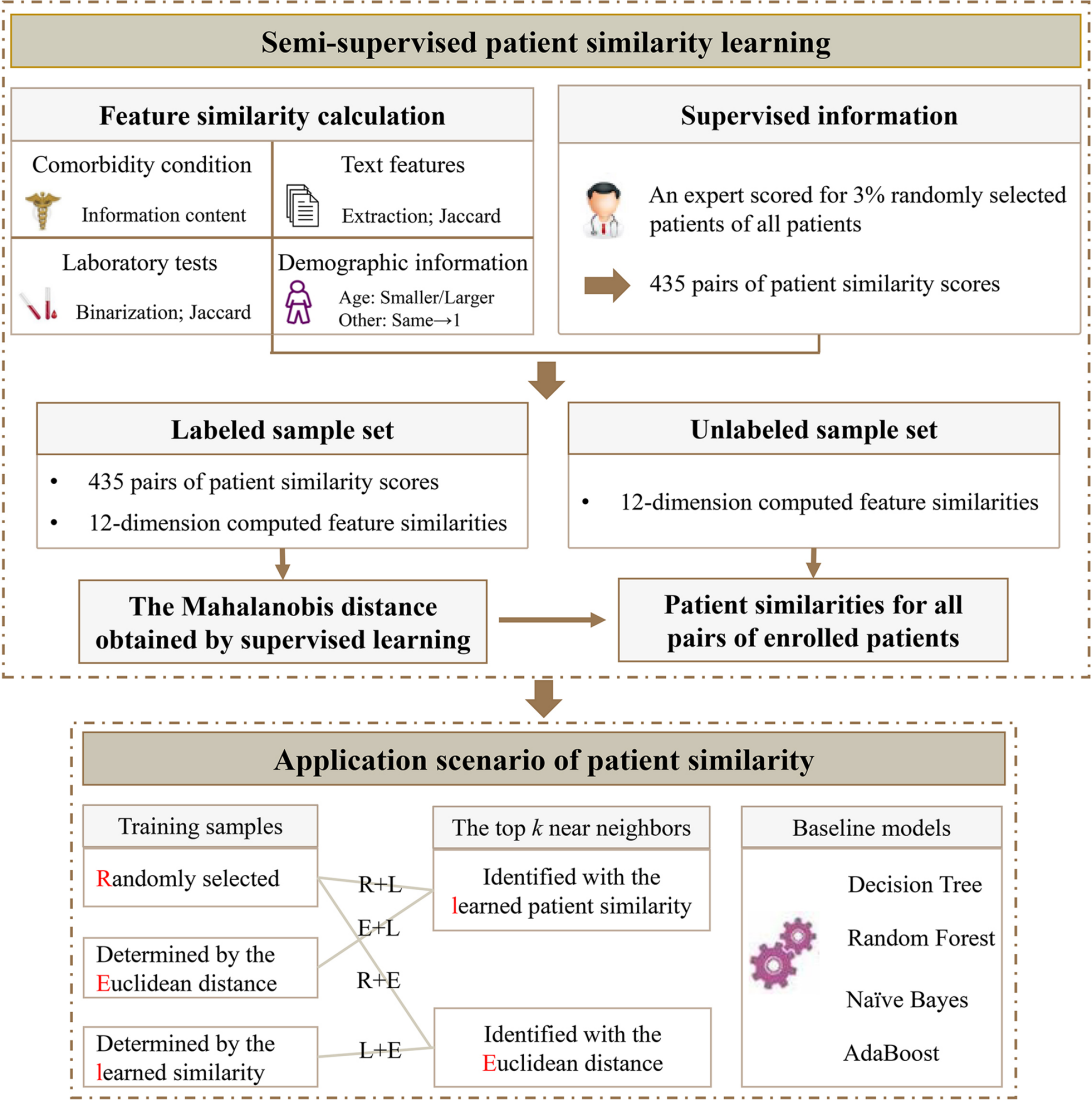
The workflow of the whole study

In this study, the proposed learning-based method included two successive steps: calculating the feature similarity directly and learning the patient similarity in a semi-supervised way. The labeled sample set, a tiny size of computed feature similarities and corresponding patient similarity scores obtained from a clinical expert, was firstly used to learn the optimal distance measurement. We then dynamically expanded the labeled sample set with a snowballing process. Figure 1 showed an overview of the whole study. Similar patients determined by the resulting similarity were finally used for identifying the prevalence of three liver diseases.

### Feature similarity calculation

In our study, EMR data consisted of four parts, demographical information, laboratory tests, comorbidity conditions, and free-text radiology reports. Similarities for the structured and unstructured, continuous and categorical features were estimated separately according to their attributes.

#### Feature similarity for comorbidity condition

**Fig. 2:**
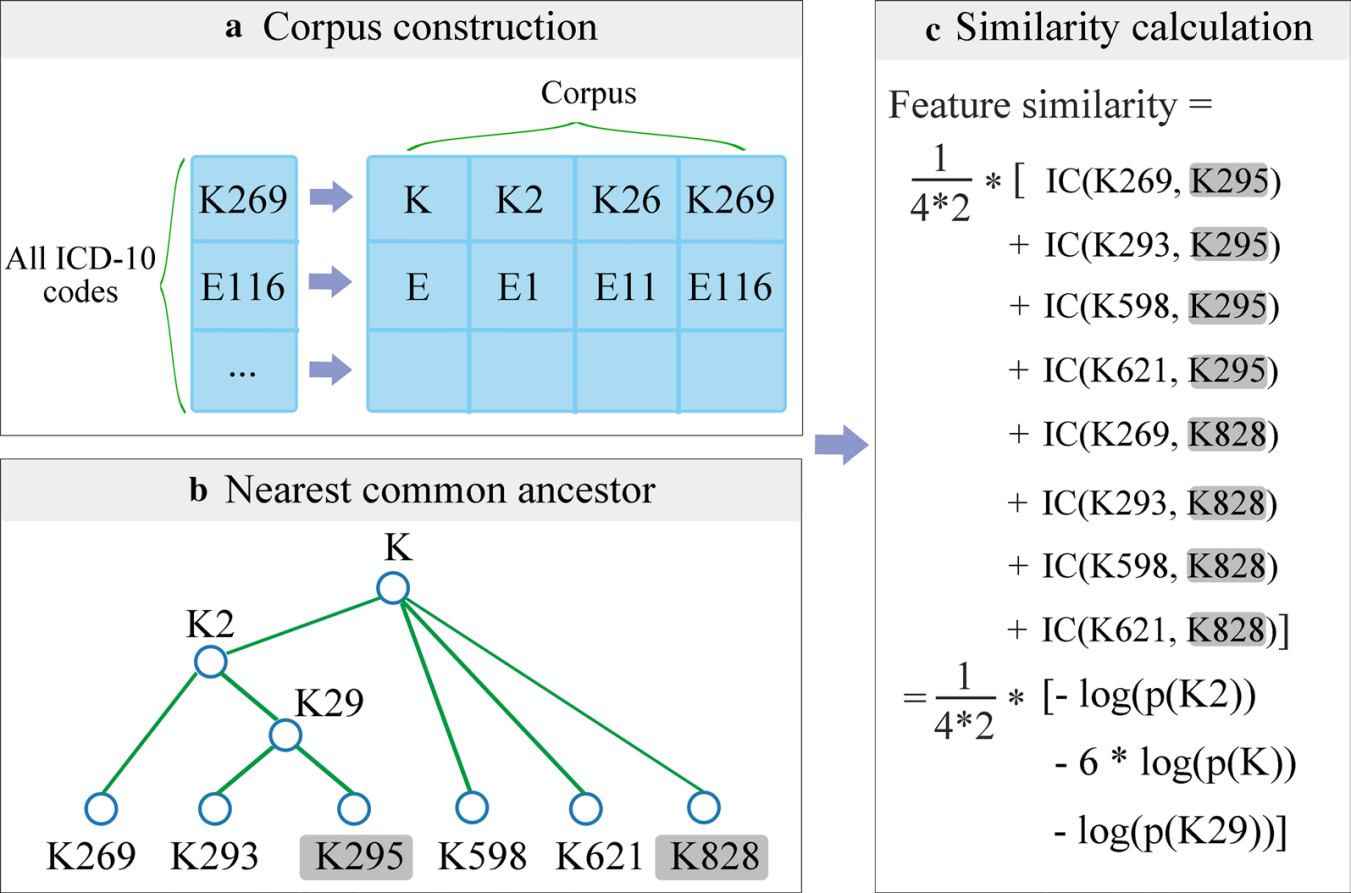
Calculation of the feature similarity for the comorbidity condition. The example patients have comorbidity conditions $\left\{ {K269, K293, K598, K621} \right\}$ and $\left\{ {K269, K293, K598, K621} \right\}$ (in shadow), respectively. **a** The pre-built study corpus. K, K2, K26, and K269 and E, E1, E11, and E116 are the qualified ICD-10 code fragments from K269 and E116, respectively; **b** identification of the nearest common ancestor (NCA). K29 is the NCA for K295 and K293, K2 for K295 and K269, and K for the rest six pairs of ICD-10 code; **c** Calculation of the comorbidity condition similarity using information content

A patient’s comorbidity conditions referred to a group of simultaneous diseases, which were identified by the International Classification of Diseases, tenth revision (ICD-10) codes [24]. An ICD-10 code began with a letter followed by five digits, arranging in a tree-like hierarchical manner [5], which was simplified into a leading letter and three following digits [5, 22] in the present study. Taking the hierarchical ontology of the ICD-10 code system into account, we measured the similarity between two ICD-10 codes by the information content (IC). IC was judged by the degree to which the two codes shared information. Two ICD-10 codes were more similar if they had a higher IC, meaning that they shared more information. The IC index for two ICD-10 codes $ICD_{1}$ and $ICD_{2}$ was defined as following [25]:

1 $$IC\left( {ICD_{1} ,ICD_{2} } \right) = IC\left( {NCA} \right) = - {\text{log}}\left( {{\text{p}}\left( {NCA} \right)} \right)$$

where NCA represented the nearest common ancestor of two codes ICD_1_ and ICD_2_, and p(NCA) was the probability of encountering the NCA in the study corpus*.* The corpus consisted of all the possible ICD-10 code fragments derived from ICD-10 codes appearing in any patient’s record in this study. An ICD-10 code fragment might be the leading letter alone or the leading letter plus the sequential one, two, or three digits of an ICD-10 code (Fig. 2a). Obviously, in the study corpus, the probability of encountering an ICD-10 code fragment that contained a leading letter K alone was not less than that of encountering K2. The larger the probability, the smaller the IC value. This result coincided with an intuitive insight that two patients were more similar if they both had the same rare disease than they both had a common flu. The IC value of two ICD-10 codes without NCA (i.e., with different leading letters) was defined as 0 because of no sharing information.

Let $X = \left\{ {ICD_{1} ,ICD_{2} , \ldots ,ICD_{m} } \right\}$ denote the comorbidity conditions for patient *i* with m comorbidities and $Y = \left\{ {ICD_{1} ,ICD_{2} , \ldots ,ICD_{n} } \right\}$ for patient *j* with n comorbidities. The feature similarity for the comorbidity condition between the two patients was defined as the following:

2 $$\frac{1}{mn}\mathop \sum \limits_{a = 1}^{m} \mathop \sum \limits_{b = 1}^{n} IC\left( {ICD_{a} ,ICD_{b} } \right)$$

where $ICD_{a} \in X$ and $ICD_{b} \in Y$. Figure 2 shows an example of calculating the feature similarity for the comorbidity condition.

#### Feature similarity for text feature

**Fig. 3:**
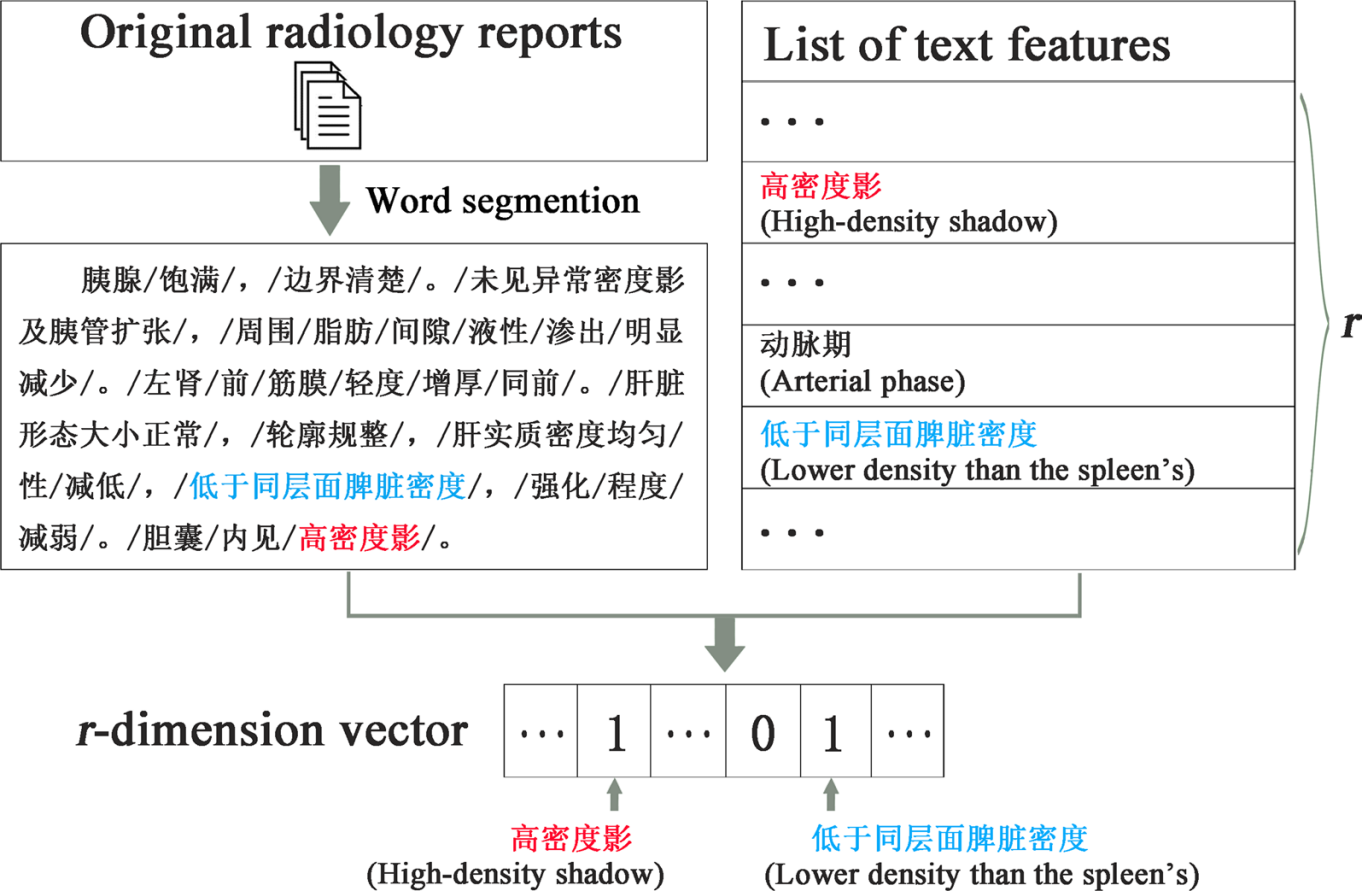
The structuration process of radiology reports

Free-text radiological reports on abdominal computed tomography (CT) stored in the EMR system were initially unstructured. Text features were recorded as words or phrases. Therefore, the radiological reports were first structured by a natural language processing (NLP) method using the term frequency-inverse document frequency (TF-IDF) score [26] to extract the important text features for the disease diagnosis. We selected *r* phrases with the highest TF-IDF scores as the most important and representative ones, and thus the diagnostically useful *r* text features for the subsequent study. Each text feature was organized as a binary variable. If a specific phrase existed within a patient’s radiological report, the patient was considered to have the corresponding text feature, and the value for this feature variable was set to 1, and 0 otherwise (Fig. 3). After the radiological report structuration, each patient had an *r*-dimension 0–1 vector of text features.

Among all the extracted text features, retroperitoneal lymph node enlargement, lower density than the spleen’s, absence of abnormal density, uniform density of liver parenchyma, and arterial phase were considered to be the most important features according to radiologists’ advice. They were then treated as independent binary features when calculating the feature similarity, while the rest were organized into a binary set of text features from the radiological reports. Feature similarity for the binary independent text features between patients *i* and *j* was defined as:

3 $$Similarity\;for\;binary\;features\left( {i,j} \right) = \left\{ {\begin{array}{*{20}l} {1,} \hfill & {if\;patients\;i\;and\;j\;had\;the\;same\;information} \hfill \\ {0,} \hfill & {otherwise} \hfill \\ \end{array} } \right.$$

The feature similarity for the text feature sets for patients *i* and *j* was defined as:

4 $$Similarity\;for\;feature\;sets\left( {i,j} \right) = \frac{{\left| {Set_{i} \cap Set_{j} } \right|}}{{\left| {Set_{i} \cup Set_{j} } \right|}}$$

#### Feature similarity for laboratory tests

We used the classification and regression trees technology to interpolate the missing values of laboratory tests, which could handle both discrete and continuous input (using the rpart function in the rpart package of R 3.5.1 software (https://cran.r-project.org/)). All the laboratory test items were binarized into 0 standing for normal or 1 for abnormal regarding the respective normal ranges. Then the feature similarity for two laboratory tests was defined as the Jaccard distance-based similarity between the two laboratory test sets.

#### Feature similarity for demographic information

Demographic characteristics included the patient’s age, sex, drug allergy history, and the source of hospital admission. We defined the feature similarity for ages as [5]:

5 $$Similarity\;for\;ages\left( {i,j} \right) = \frac{{min\left( {{\text{Age}}_{i} ,{\text{Age}}_{j} } \right)}}{{max\left( {{\text{Age}}_{i} ,{\text{Age}}_{j} } \right)}}$$

The similarity for binary features, including sex, drug allergy history, and source of hospital admission between patients *i* and *j* was defined as 1 if the two patients had the same information and 0 otherwise [Eq. (3)].

In total, four demographic features, namely, age, sex, drug allergy history, and the source of hospital admission, five text features specific to the predictive task, and three feature sets, i.e., comorbidity conditions, laboratory tests, and common text features from radiological reports, were involved in the calculation for the feature-level similarity. A resulting 12-dimension similarity vector was generated for each pair of patients.

### Semi-supervised patient similarity learning

In this study, we randomly selected a tiny fraction of the overall patients. An experienced domain medical expert provided a similarity score ranging from 0 to 1 for each of possible pairs among these patients. We also calculated the weighted sum of feature similarities as the patient similarity for each patient pair. The weights were determined through trial and error. The expert could adjust his score with reference to the calculated patient similarity. A patient pair with both the feature similarity vector (calculated in section *Feature similarity calculation*) and the patient similarity score (assigned by the expert) was considered as a labeled sample. Thus the labeled sample set consisted of all labeled samples, and the patient pairs without any known similarity score comprised the unlabeled sample set.

Further, a continuous label was transformed into a new categorical label “constraint.” Constraint values of *must-link*, *general-link*, and *cannot-link* were given to patient pairs with higher, median, and lower similarity scores, respectively. The cutoff points of similarity scores were set to the upper and lower tertiles. Patient pairs with the same constraint belonged to the same class. We used both continuous and categorical labels in the semi-supervised similarity learning.

Based on the labeled sample set, our goal was to learn a Mahalanobis distance between a patient pair. For a patient pair of $x_{i}$ and $x_{j}$, the Mahalanobis distance $d_{m} \left( {x_{i} ,{ }x_{j} } \right)$ was defined as

6 $$d_{m} \left( {x_{i} ,{ }x_{j} } \right) = \sqrt {\left( {x_{i} - x_{j} } \right)^{T} C\left( {x_{i} - x_{j} } \right)}$$

where *C* $\in R^{d \times d}$ (*d* = 12 as described in section *Feature similarity for demographic information*) was a positive semi-definite covariance matrix. The learning aimed to get the optimal *C*, which could minimize the within-class squared distances and maximize the between-class squared distances among all patient pairs on the labeled sample set, simultaneously. We firstly constructed a goal function with the ratio of the with-class squared distances and the between-class squared distances, then transformed the learning problem into a trace quotient minimization problem with matrix decomposition. Finally, we solved this trace quotient minimization problem with the decomposed Newtown’s method [27]. The optimization of matrix C on the labeled sample set was a supervised learning process. See supplementary materials for more details and descriptions about the learning process.

Once the matrix C was fixed, it was used to calculate the Mahalanobis distances [Eq. (6)] between the labeled and unlabeled samples. A small batch of unlabeled samples obtained similarity labels from their nearest labeled neighbors measured by Mahalanobis distances, and became members of the labeled sample set. Then the dynamically expanded labeled sample set was used for providing near neighbors for the next batch of unlabeled samples. The process was repeated until all unlabeled samples got a similarity label. We set the batch size to 1153 (about 0.15% samples of all the unlabeled samples) in each round based on trial and error.

### Building predictive models

Building and assessing a similarity- or distance-based classifier was considered feasible and suitable for validating the effectiveness of the proposed similarity. In this study, a *k*NN model was employed for a multi-class classification task. By trial and error, the optimal *k* was set to 10 with which the corresponding *k*NN model built on the whole training samples showed the best predictive performance in the application scenario. When applying this model, we had many choices concerning the composition and size of the training samples and the similarity measurement used in determining near neighbors, which might all cause differences in the predictive performance. We used a leave-one-out method to evaluate the performances of the predictive models. In each validation round, one patient in overall patients was used as a test sample and the rest patients comprised the training sample pool. The training samples for an *k*NN classifier were M% (M ranging from 2 to 100) samples selected from this training sample pool. The training samples might be randomly selected from the pool or selected with the learned similarity or Euclidean distance to help rule out some irrelevant samples. The proposed similarity and Euclidean distance were also used to determine near neighbors. We evaluated and compared the proposed patient similarity with respects of i) the selection of the training samples from the training sample pool; ii) the determination of the *k* nearest neighbors; and iii) the usage of patient similarity. For the first two, we had four combinations as follows.

- *R* + *L combination:* the training samples were randomly selected from the training sample pool, and the top *k* nearest neighbors were identified with the learned patient similarity;
- *R* + *E combination:* the training samples were randomly selected from the training sample pool, and the top *k* nearest neighbors were identified with Euclidean distance;
- *L* + *E combination:* the training samples were the most similar samples determined by the learned similarity, and the top *k* nearest neighbors were identified with Euclidean distance;
- *E* + *L combination:* the training samples were the most similar samples determined by Euclidean distance, and the top *k* nearest neighbors were identified with the learned similarity;

As a typical type of non-numeric variables, comorbidity conditions were not suitable for calculating the Euclidean distance-based similarity directly. Thus, we selected 26 popular comorbidities with an occurrence greater than 5% among all patients, each being treated as a binary variable. Finally, in the Euclidean distance-related predictive models (combinations R + E, L + E, and E + L), an input sample had 131 features: 44 features were the most important and representative phrases extracted from the radiological reports as the text features; 57 features were regular laboratory test items such as blood tests and urine tests; 26 features were comorbidity variables such as diabetes mellitus with and without complications, and essential hypertension; and the rest four features were demographic variables. The predicted label for an index patient was assigned as the label of the majority of his *k* nearest neighbors.

Besides, we compared the performances between personalized *k*NN models and other state-of-the-art machine learning models without using patient similarity as references, including decision trees, Naïve Bayes classifier, random forest, and AdaBoost classifier integrating weak decision trees. The leave-one-out validation on the whole training sample pool was also used.

The performance metric included the micro-averaged area under the receiver operating characteristic curve (AUC), F1-score, and cross-entropy (CE) loss [28]. We used the cubic polynomial fitting to the changing trends of the three metrics, respectively.

### Data set

EMR data used in this study were derived from inpatients discharged from a tertiary hospital in Beijing, China between 2014 and 2016. Individual hospitalizations were de-identified and maintained as unique records, including age at admission, sex, drug allergy history, source of hospital admissions, up to 11 comorbidities at discharge, laboratory tests, and radiology reports during hospitalization.

Because only radiology reports of CT examination were available at present, 6749 patients who underwent liver CT scans were enrolled in this study. Liver cancer, hemangioma, and NAFLD were determined according to the radiological impression section in a radiological report and were further confirmed by ICD-10 codes in the comorbidity fields of a patient’s record. ICD-10 codes for liver cancer, hemangioma, and NAFLD were C22 (malignant neoplasm of liver and intrahepatic bile ducts) and C78.7 (secondary malignant neoplasm of liver and intrahepatic bile duct), D18.0 (hemangioma, any site), and K76.0 (nonalcoholic fatty liver disease), respectively. Following the above criteria, we finally identified 153, 178, and 403 patients diagnosed with liver cancer, hemangioma, and NAFLD correspondingly. Together with 449 patients with a normal liver diagnosis, records of these 1183 patients comprised the study dataset. Finally, we excluded the ICD-10 codes of C22, C78.7, D18.0, and K76.0 that were used to identify the target diseases from the comorbidity conditions for computing the feature similarity. Table 1 gives some details of the study population.

**Table 1:** Some basic characteristics of patients with liver cancer, hemangioma, non-alcohol fatty liver, and normal liver diagnosis

Characteristic	Liver cancer (n = 153)	Hemangioma (n = 178)	Non-alcohol fatty liver (n = 403)	Normal (n = 449)
Demographic information				
Age, year, mean ± standard deviation	66.6 ± 0.93	57.2 ± 0.85	51.4 ± 0.68	51.8 ± 0.63
Male gender, n (%)	101 (66.0)	85 (47.8)	233 (57.8)	228 (50.8)
Drug allergy history, n (%)	13 (8.5)	23 (12.9)	80 (19.9)	58 (12.9)
Outpatient admission, n (%)	130 (85.0)	164 (92.1)	321 (79.7)	407 (90.6)
Comorbidity condition, n (%)				
Coronary heart disease	13 (8.5)	10 (5.6)	27 (6.7)	31 (6.9)
Diabetes mellitus without complication	28 (18.3)	18 (10.1)	90 (22.3)	57 (12.7)
Diabetes mellitus with complication	7 (4.6)	8 (4.5)	44 (10.9)	21 (4.7)
Essential hypertension	61 (39.9)	61 (34.3)	169 (41.9)	116 (25.8)
Disorders of lipid metabolism	3 (2.0)	21 (11.8)	135 (33.5)	71 (15.8)
Other gastrointestinal disorders	23 (15.0)	31 (17.4)	61 (15.1)	67 (14.9)
Laboratory test, n (%)				
Abnormal urine leukocytes	13 (8.5)	17 (9.6)	37 (9.2)	34 (7.6)
Abnormal urine bilirubin	4 (2.6)	2 (1.1)	8 (2.0)	9 (2.0)
Abnormal urobilinogen	9 (5.9)	3 (1.7)	12 (3.0)	17 (3.8)
Abnormal urine glucose	9 (5.9)	14 (7.9)	56 (13.9)	42 (9.4)
Abnormal urine occult blood	20 (13.1)	18 (10.1)	25 (6.2)	37 (8.2)
Abnormal ketone	12 (7.8)	16 (9.0)	38 (9.4)	31 (6.9)

## Results

### Patient similarity

We randomly selected 30 patients out of the 1183 study patients to construct the labeled set with 435 samples (patient pairs). The rest 698,718 (= 1183 * (1183–1)/2–435) samples comprised the unlabeled sample set. Among the 30 patients, four patients were with liver cancer, five with hemangioma, ten with NAFLD, and 11 with a normal liver diagnosis, retaining the same disease constituent ratio for the labeled set as that for the whole study population. There were statistically differences in similarity scores (mean ± standard deviation: 0.77 ± 0.082, 0.54 ± 0.043, and 0.37 ± 0.074, respectively; one-way analysis of variance, all P values < 0.001 after the Bonferroni adjustment) among the patient pairs with the constraints of *must-link*, *general-link*, and *cannot-link*. See supplementary materials for some examples of patient pairs with different similarity scores (Additional file 1: Table S1).

**Fig. 4:**
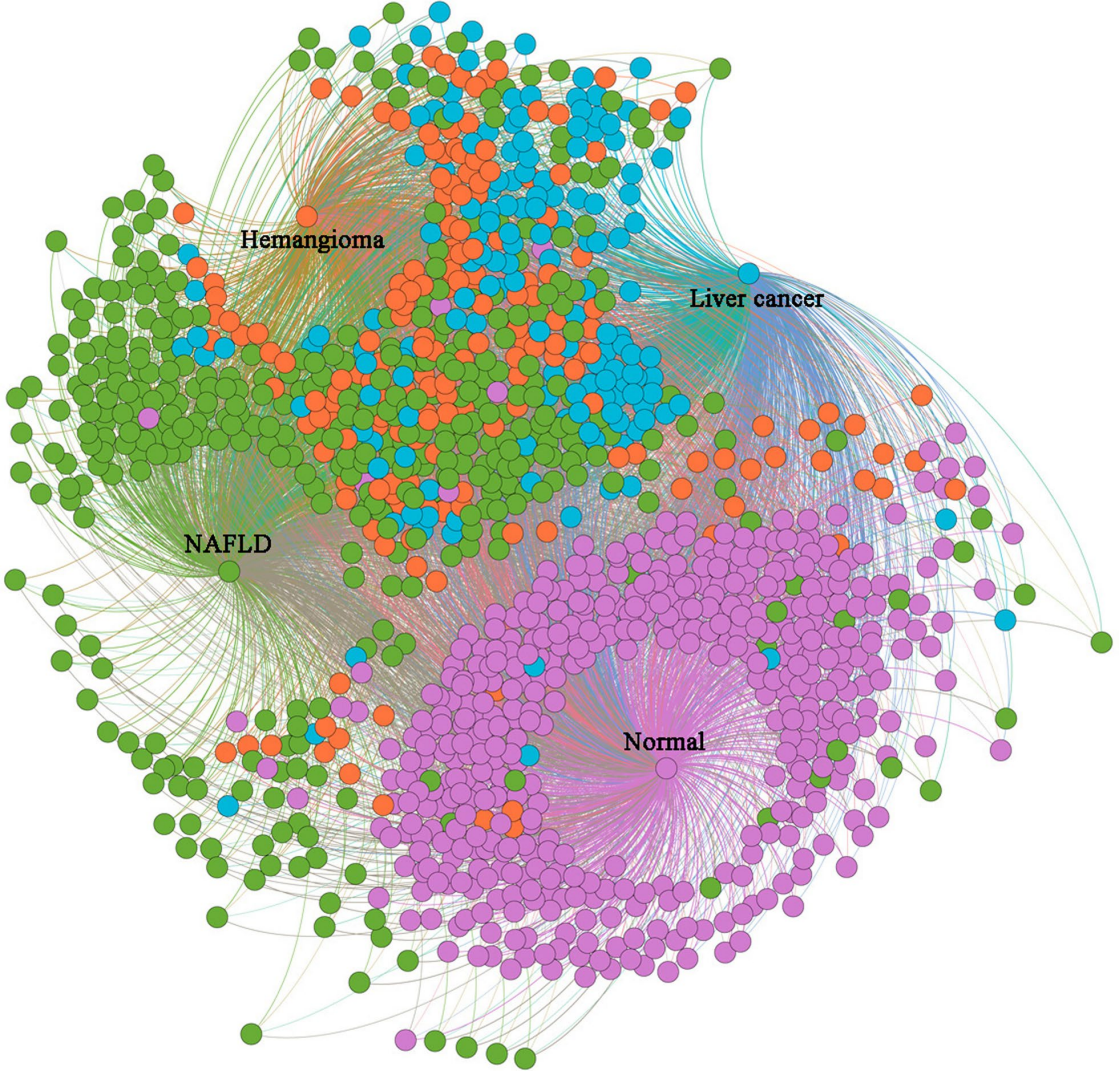
Illustration of the learned patient similarity. Four index patients (the central dots) are surrounded by all other patients, where the more similar a patient is with the index patient, the closer the respective dot is to the central dot. Dots in blue, orange, green, and purple represent patients with liver cancer, hemangioma, NAFLD, and normal condition, respectively. The figure was generated by using Gephi 0.9.2 (https://gephi.org/). NAFLD, non-alcohol fatty liver disease

We obtained patient similarities for all the patient pairs among the overall 1183 patients after the semi-supervised learning. Figure 4 is a visualized example of the learned patient similarities between four randomly selected patients (each with one of the four liver conditions) and the rest.

### Disease prediction

**Fig. 5:**
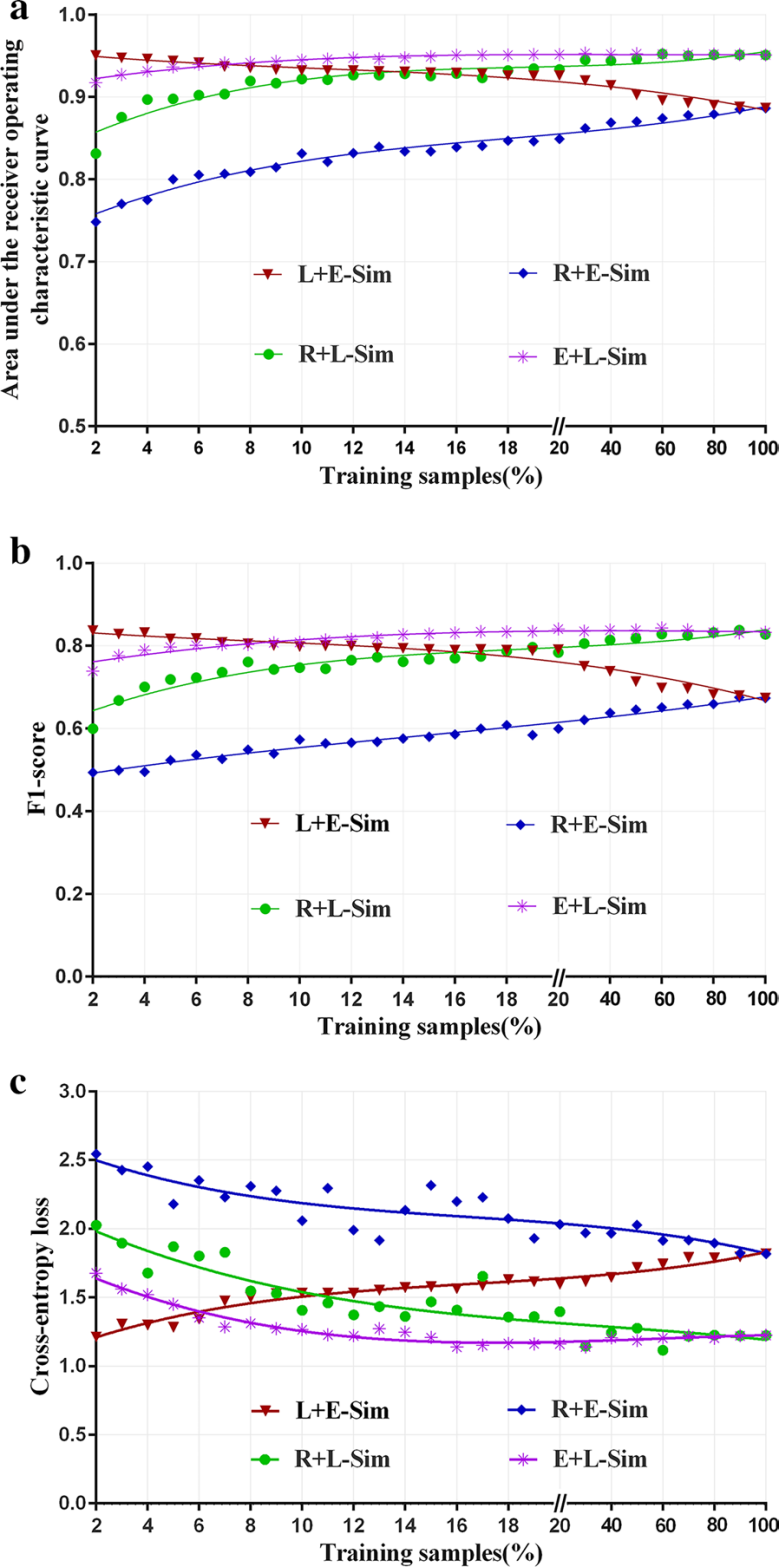
Performance of k-nearest neighbor models in terms of **a** area under the receiver operating characteristic curve, **b** F1-score, and **c** cross-entropy loss. The horizontal axes represent the sizes of the training samples in percentage. R + L combination and R + E combination, the *k*NN models that the nearest neighbor out of the randomly selected training samples was determined by the learned similarity and the Euclidean distance, respectively; L + E combination, the *k*NN model that the nearest neighbor out of the similar training samples based on the learned similarity was determined by the Euclidean distance; E + L combination, the *k*NN model that the nearest neighbor out of the similar training samples based on the Euclidean distance was determined by the learned similarity

As the size of training samples used for building *k*NN models increased from 2% (about 23 patients) to 100% (1182 patients) of the whole study samples, the predictive performance of the L + E combination degraded gradually from the initial values of 0.95 and 0.84 to the values of 0.89 and 0.67 in terms of AUC and F1-score, respectively, while the CE loss increased from 1.21 to 1.82. Contrary to the predictive performance of the L + E combination, those of the E + L, R + L, and R + E combinations gradually increased from 0.92 and 0.74, 0.83 and 0.60, and 0.75 and 0.49 to 0.95 and 0.84, 0.95 and 0.84, and 0.89 and 0.67 in terms of AUC and F1-score, separately, while the CE loss decreased from 1.68, 2.03, and 2.54 to 1.22, 1.22, and 1.82, separately (Fig. 5).

There were significantly higher performances of models based on the learned patient similarity (i.e., L + E combination, E + L combination, and R + L combination) than those based on the directly calculated patient similarity (i.e., R + E combination) (Kruskal Wallis Tests, P values < 0.001 for all performance indexes). When using no more than 7% (about 82 patients) of the whole training samples, the L + E combination outperformed the E + L combination. Based on all training samples, the performance of the L + E combination and R + E combination showed the same performance of AUC 0.89, F1-score 0.67, and CE loss 1.82. Simultaneously, the E + L combination and R + L combination showed the same performance of AUC 0.95, F1-score 0.84, and CE loss 1.22, respectively.

Among the four reference models, the highest AUC and F1-score were 0.94 and 0.80, separately, which were both lower than those for the simple and similarity-based kNN models (Table 2).

**Table 2:** Comparison of predictive performance between similarity-based models with other machine learning models

	Area under the receiver operating characteristic curve	F1-score	Cross-entropy loss
Naive bayes	0.92	0.80	3.60
Decision tree	0.91	0.73	*0.72*
Random forest	0.94	0.78	0.75
AdaBoost	0.86	0.70	1.16
R + E/L + E combination	0.89	0.67	1.84
R + L/E + L combination	*0.95*	*0.84*	1.23

## Discussion

In personalized medicine, using machine learning algorithms and patient similarity based on real-world EMR data had significant facilitation in various scenarios such as disease prediction [5–7] and risk factors identification [8, 9]. However, EMR data usually had mixed data types, structured and unstructured, and continuous and categorical, making it a challenge to measure the similarity among patients. Under this situation, the learning-based patient similarity measurement was proposed and evaluated in this study.

When computing the feature-level similarity for two patients, we employed different similarity measurements according to the features’ attributes (data types). Besides the relative ratio for continuous feature (age) and Jaccard similarity index for binary features (binarized laboratory test results, text features, and other demographic characteristics), IC measure was applied to the computation of the feature similarity for the ICD-10 code-based comorbidity conditions. As a coded and hierarchical feature, ICD-10 code could be considered either as a general multi-categorical variable thus using the Jaccard distance to measure their similarities [11, 22, 29], or as a tree-like variable thus using a path-based measurement to estimate their similarities with the full use of the hierarchical information [25]. Among the path-based measurements, the edge-counting approach had been used in many previous studies [3, 5, 30]. Two patients diagnosed with liver cancer of different ICD-10 codes (C22.0 and C22.9, for example) had the same similarity as those diagnosed with influenza of different ICD-10 codes (J11.1 and J11.2, for example), both having the same edge count 3. However, the former patient pair should be considered more similar than the latter because liver cancer was less common in the general population than the flu. As an alternative path-based measurement, IC had the advantage of dealing with this problem. It was a probabilistic model [25] and had been applied to the gene ontology terms [31, 32] and semantic context [33–35]. To our best knowledge, it was the first time to use the corpus-based IC measurement to measure the similarity for the comorbidity conditions based on ICD-10 codes.

Except for the out-of-the-box patient features, text features that were extracted from free-text radiological reports were also involved in the calculation of the feature-level similarity in the current study. We firstly used an NLP method to extract features. All features had the same contribution to the Jaccard distance-based similarity, no matter how important they were to the target disease. However, the situation was not the same in radiological practice. Thus, the extracted features were then structured into one binary feature vector and five independent binary features at the radiologist’s suggestion to highlight the role they played on the similarity calculation. This was a simple but successful attempt to add structured text features into the similarity calculation.

Another critical factor in getting a high-quality patient-level similarity was the way to consolidate the feature-level similarities. Instead of a linear or logarithmic combination of the individual feature similarities [3, 5, 12], we proposed a semi-supervised learning process in our study. For learning tasks, sufficient supervised information was crucial and desired, whereas annotation of EMRs required sophisticated medical professionals, which was very time-consuming and expensive [36]. Thus, the SSL algorithm based on a small amount of supervised information was used by numerous researchers in the ticket classification problem [37], the image segmentation problem [38], and the phenotype stratification problem [39] and so on. In this study, the SSL algorithm was adopted with a tiny labeled fraction of the overall patients to balance the learning performance and label-requested difficulties.

Previous studies have proved that similarity-based predictive models outperformed those without sample selection according to patient similarity [5, 8, 22]. In this study, even though we only labeled 2.5% of the study population for the semi-supervised learning, the learned patient similarity still played a significant role in improving the similarity-based *k*NN models’ predictive performances. The personalized *k*NN models outperformed other state-of-the-art machine learning models without using patient similarity, which demonstrated the effectiveness and superiority of the proposed patient similarity measurement. Because the *k*NN classification method used the *k* nearest neighbors to vote for the output label, the way to determine the nearest neighbor surely did matter. The *k*NN models using the learned patient similarity to determine near neighbors consistently outperformed those using the Euclidean distance to determine near neighbors. This indicated that using the proposed similarity could find out more similar patients than using the widely used similarity measurement, the Euclidean distance. Furthermore, even if the simple Euclidean distance was used to determine near neighbors, the learned patient similarity could still improve the predictive performance by helping select the similar training samples from which the *k* nearest neighbors were selected. However, the performance of this kind of *k*NN model degraded gradually against the size of the training samples, coinciding with the previous studies’ conclusion that adding more dissimilar samples would disturb the prediction [5, 8]. The decreasing trend also indicated that as the size of the training samples increased, more dissimilar and irrelevant samples would be added, leading to the Euclidean distance's weakened ability to retrieve the most similar sample. On the contrary, predictive performances increased gradually when near neighbors determined with the learned patient similarity was selected from similar training samples identified with the Euclidean distance, showing the strength of the learned patient similarity in selecting near neighbors even from more dissimilar samples. In short, the proposed learning-based method successfully dealt with the problem when measuring the similarity of patients stored in heterogeneous EMRs.

This study had a few limitations. First, the determination of patient features for computing similarities depended partially on domain knowledge and aimed at a specific predictive task. There still need an effort to generalize the proposed patient similarity to other application scenario and design an adaptive feature selection. Second, only one expert was asked to score the patient similarities in this study. Though the human expert could give scores with the assistance of the calculated patient similarity, this scoring mechanism might exist labeling bias. Finally, considering the labor cost, we only labeled 30 randomly selected patients (generating 435 patient pairs) manually. To obtain similarity labels for hundreds and thousands of patient couples efficiently and effectively, we will further integrate active learning into the proposed semi-supervised learning method.

## Conclusions

In this study, we proposed a semi-supervised learning method to obtain patient similarity using heterogeneous EMR data. It has proved that the similar samples identified with the proposed similarity measurement would be helpful to improve the performance of the similarity-based predictive models. The proposed method is expected to be used in other heterogeneous EMR data for machine learning tasks that originated from clinical practice.

## Supplementary Information

**Additional file 1.** Details of the semi-supervised learning method and examples of the labeled samples.

## Data Availability

Not applicable.

## Abbreviations

AUC: Area under the receiver operating characteristic curve
CE: Cross-entropy
CT: Computed tomography
EMR: Electronic medical record
IC: Information content
ICD-10: International Classification of Diseases, the tenth revision
*k*NN: K-nearest neighbor
NAFLD: Non-alcohol fatty liver disease
NCA: Nearest common ancestor
NLP: Nature language processing
SSL: Semi-supervised learning
TF-IDF: Term frequency-inverse document frequency

## References

[1] Sharafoddini A, Dubin JA, Lee J (2017). Patient similarity in prediction models based on health data: a scoping review. JMIR Med Inform.

[2] Parimbelli E, Marini S, Sacchi L (2018). Patient similarity for precision medicine: a systematic review. J Biomed Inform.

[3] Gottlieb A, Stein GY, Ruppin E (2013). A method for inferring medical diagnoses from patient similarities. BMC Med.

[4] Wu J, Roy J, F. SW, (2010). Prediction modeling using EHR data_challenges, strategies, and a comparison of machine learning approaches. Med Care.

[5] Wang N, Huang Y, Liu H (2019). Measurement and application of patient similarity in personalized predictive modeling based on electronic medical records. Biomed Eng Online.

[6] Henriques J, Carvalho P, Paredes S (2015). Prediction of heart failure decompensation events by trend analysis of telemonitoring data. IEEE J Biomed Health Inform.

[7] Guttag J, Syed Z (2011). Unsupervised similarity-based risk stratification for cardiovascular events using long-term time-series data. J Mach Learn Res.

[8] Ng K, Sun J, Hu J (2015). Personalized Predictive Modeling and Risk Factor Identification using Patient Similarity. AMIA Summits Transl Sci Proc.

[9] Wang X, Wang F, Wang J, et al. Exploring patient risk groups with incomplete knowledge. In: 2013 IEEE international conference on data mining (ICDM). IEEE; 2013. p. 1223–1228.

[10] Li L, Cheng WY, Glicksberg BS (2015). Identification of type 2 diabetes subgroups through topological analysis of patient similarity. Sci Transl Med.

[11] Shu Z, Liu W, Wu H (2018). Symptom-based network classification identifies distinct clinical subgroups of liver diseases with common molecular pathways. Comput Methods Progr Biomed.

[12] Gottlieb A, Stein GY, Ruppin E (2011). PREDICT: a method for inferring novel drug indications with application to personalized medicine. Mol Syst Biol.

[13] Xiao J, Wang F, Wong N-K (2019). Global liver disease burdens and research trends: analysis from a Chinese perspective. J Hepatol.

[14] Adibi A, Maleki S, Adibi P (2017). Prevalence of nonalcoholic fatty liver disease and its related metabolic risk factors in Isfahan. Iran Adv Biomed Res.

[15] Zhang W, Huang ZY, Ke CS (2015). Surgical treatment of giant liver hemangioma larger than 10 cm: a single center's experience with 86 patients. Medicine (Baltimore).

[16] Hoekstra LT, Bieze M, Erdogan D (2013). Management of giant liver hemangiomas: an update. Expert Rev Gastroenterol Hepatol.

[17] Bray F, Ferlay J, Soerjomataram I (2018). Global cancer statistics 2018: GLOBOCAN estimates of incidence and mortality worldwide for 36 cancers in 185 countries. CA Cancer J Clin.

[18] Dai L, Zhu H, et al. Patient similarity: methods and applications. 2020. https://arxiv.org/abs/2012.01976. Accessed 5 Dec 2020.

[19] Lee J, Maslove DM, Dubin JA (2015). Personalized mortality prediction driven by electronic medical data and a patient similarity metric. PLoS ONE.

[20] David G, Bernstein L, Coifman RR (2011). Generating evidence based interpretation of hematology screens via anomaly characterization. Open Clin Chem J.

[21] Gu D, Liang C, Zhao H (2017). A case-based reasoning system based on weighted heterogeneous value distance metric for breast cancer diagnosis. Artif Intell Med.

[22] Huang Y, Wang N, Liu H (2019). Study on patient similarity measurement based on electronic medical records. Stud Health Technol Inform.

[23] Jia Z, Zeng X, Duan H (2020). A patient-similarity-based model for diagnostic prediction. Int J Med Inform.

[24] ICD-10 Version. 2019. https://icd.who.int/browse10/2019/en#/. Accessed 20 Aug 2020.

[25] Popescu M, Xu D. Data mining in biomedicine using ontologies. Artech House. 2009.

[26] Salton G, McGill MJ (1983). Introduction to modern information retrieval.

[27] Jia Y, Nie F, Zhang C (2009). Trace ratio problem revisited. IEEE Trans Neural Networks.

[28] Bishop CM (2006). Pattern recognition and machine learning (information science and statistics).

[29] Girardi D, Wartner S, Halmerbauer G (2016). Using concept hierarchies to improve calculation of patient similarity. J Biomed Inform.

[30] Popescu M, Khalilia M (2011). Improving disease prediction using ICD-9 ontological features. IEEE Int Conf Fuzzy Syst.

[31] Mazandu GK, Mulder NJ (2013). DaGO-Fun: tool for Gene Ontology-based functional analysis using term information content measures. BMC Bioinform.

[32] Milano M, Agapito G, Guzzi PH (2016). An experimental study of information content measurement of gene ontology terms. Int J Mach Learn Cybern.

[33] Sánchez D, Batet M (2011). Semantic similarity estimation in the biomedical domain: An ontology-based information-theoretic perspective. J Biomed Inform.

[34] Kamoun K, Yahia SB. Stability assess based on enhanced information content similarity measure for ontology enrichment. In: International conference on model and data engineering. 2014.

[35] Milne D, Witten IH (2013). An open-source toolkit for mining Wikipedia. Artif Intell.

[36] Wang F (2015). Adaptive semi-supervised recursive tree partitioning: The ART towards large scale patient indexing in personalized healthcare. J Biomed Inform.

[37] Wang F, Sun J, Li T, et al. Two heads better than one: metric + active learning and its applications for IT service classification. In: ICDM 2009, proceedings of the 2009 ninth IEEE international conference on data mining. 2009. p. 1022–1027.

[38] Bai W, Oktay O, Sinclair M, Descoteaux M, Maier-Hein L, Franz A (2017). Semi-supervised learning for network-based cardiac MR image segmentation. Medical image computing and computer-assisted intervention—MICCAI 2017.

[39] Beaulieu-Jones BK, Greene CS (2016). Semi-supervised learning of the electronic health record for phenotype stratification. J Biomed Inform.

