# Supplementary material for: Study on the semi-supervised learning-based patient similarity from heterogeneous electronic medical records
Source: BMC Med Inform Decis Mak. 2021 Jul 30;21(Suppl 2):58. doi: 10.1186/s12911-021-01432-x (PMC8323210; doi:10.1186/s12911-021-01432-x)
Supplement: Supplementary file 1 — Additional file 1. Details of the semi-supervised learning method and examples of the labeled samples. [file 12911_2021_1432_MOESM1_ESM.docx]

**Semi-supervised learning method:**

Based on the labeled sample set, our goal was to learn a Mahalanobis distance between a patient pair. For a patient pair of $x_{i}$ and $x_{j}$, the Mahalanobis distance was defined as

| $d_{m}\left( x_{i}, x_{j} \right)=\sqrt{{(x_{i}-x_{j})}^{T}C(x_{i}-x_{j})}$ | (1) |
| --- | --- |

where *C* $\in R^{d\times d}$ (*d*=12 as described in section 2.1.4) was a positive semi-definite covariance matrix. The aim of learning was to get the optimal *C*, which could minimize the within-class squared distances and maximize the between-class squared distances among all patient pairs, simultaneously. The goal function *γ* was defined as

| $\gamma= \frac{\sum_{i} \sum_{j:x_{j}\in N_{i}^{o}} {(x_{i}-x_{j})}^{T}C(x_{i}-x_{j})}{\sum_{i} \sum_{k:x_{k}\in N_{i}^{e}} {(x_{i}-x_{k})}^{T}C(x_{i}-x_{k})}$ | (2) |
| --- | --- |

where $N_{i}^{o}$ was the homogeneous set of *x_i_*’s nearest neighbors whose constraints were the same as *x_i_*’s, and $N_{i}^{e}$ was the heterogeneous set of *x_i_*’s nearest neighbors whose constraints were different from *x_i_*’s^[1]^. A data point was considered as the nearest neighbor of $x_{i}$ if the Euclidean distance among the data point and the surrounding neighbors was the shortest^[2]^. The sizes of homogeneous and heterogeneous neighborhoods were both set to 10 in this study. To avoid solving up to *d*(*d*+1)/2 variables in Eq.(2) to minimize *γ*^[1]^, the matrix *C* was decomposed to

| $C = WW^{T}$ | (3) |
| --- | --- |

where W was a *d*×2 matrix.

Substituting the Eq.(3) into the Eq.(2), we got the following optimization problem:

| ${min}_{W} \frac{tr(W^{T}M_{c}W)}{tr(W^{T}M_{S}W)}$ | (4) |
| --- | --- |

where tr(·) denotes the matrix trace, and Mc and Ms were the compactness and scatterness matrices, respectively:

| $M_{C} = \sum_{i} \sum_{j:x_{j}\in N_{i}^{o}} {(x_{i}-x_{j})}^{T}(x_{i}-x_{j})$ | (5) |
| --- | --- |
| $M_{S} = \sum_{i} \sum_{k:x_{k}\in N_{i}^{e}} {(x_{i}-x_{k})}^{T}(x_{i}-x_{k})$ | (6) |

We used the decomposed Newtown’s method^[3]^ to solve this trace quotient minimization problem and finally got the learned covariance matrix C in Eq.(1).

**References**

[1] Wang F, Sun J, Li T, et al. Two Heads Better Than One: Metric+Active Learning and its Applications for IT Service Classification. In: ICDM 2009, Proceedings of the 2009 Ninth IEEE International Conference on Data Mining, 2009: 1022-1027.

[2] Wang F , C Z. Feature Extraction by Maximizing the Average Neighborhood Margin. In: 2007 IEEE Conference on Computer Vision and Pattern Recognition, Minneapolis, MN, 2007: pp. 1-8.

[3] Jia Y, Nie F, Zhang C. Trace ratio problem revisited[J]. IEEE Transactions on Neural Networks, 2009, 20(4): 729-735.

**Examples of similarity scoring:**

Table S1 Examples of patient pairs with different similarity scores

| Patient pair | Description for the two patients | Calculated similarity | Similarity score ranked manually |
| --- | --- | --- | --- |
| Patients A and B | Patient A was 40 years old, and B was 77. They were of different gender and drug allergy history, but the same source of hospital admission.  Patient A was diagnosed with fatty liver with the comorbidities of diabetes, atherosclerosis of arteries, cardiac arrhythmia, and tachycardia. The other was diagnosed with a normal liver condition with the comorbidities of hypertension and the cecum's malignant neoplasm.  Patient A's radiological report described that liver parenchyma density was lower than that of the spleen, while patient B was uniform.  Patient A's serum carcinoembryonic antigen was normal, while patient B was abnormal. | 0.115 | 0.115 |
| Patients C and D | Patient C was 71 years old, and patient D was 50. They were of different gender and drug allergy history, but the same source of hospital admission.  Patient C was diagnosed with hemangioma with the comorbidities of duodenal ulcer, chronic superficial gastritis, other specified functional intestinal disorders, and rectal polyp. The other was diagnosed with fatty liver with the comorbidities of hypertension, paroxysmal atrial fibrillation, and hyperuricemia.  Patient C’s radiological report described that liver parenchyma density was low, while the liver parenchyma’ density of patient B was uniform. | 0.516 | 0.500 |
| Patients E and F | Patient E and F were of the same gender and age (61 years), drug allergy history, and the source of hospital admission.  The two patients were both diagnosed as normal and shared comorbidities of atherosclerotic heart disease and myocardial infarction.  The two patients’ radiological reports were almost the same. | 0.893 | 0.900 |
